# Calcitonin-producing well-differentiated neuroendocrine carcinoma (carcinoid tumor) of the urinary bladder: case report

**DOI:** 10.1186/1471-2407-5-88

**Published:** 2005-07-27

**Authors:** Massimo Mascolo, Vincenzo Altieri, Chiara Mignogna, Giorgio Napodano, Gaetano De Rosa, Luigi Insabato

**Affiliations:** 1 Department of Biomorphological and Functional Sciences, Pathology Section, University of Naples "Federico II", via S. Pansini 5, 80131, Naples, Italy; 2 Department of Urology, University of Naples "Federico II", via S. Pansini 5, 80131, Naples, Italy

## Abstract

**Background:**

The occurrence of calcitonin-secreting primary carcinoid tumor of the urinary bladder is extremely rare.

**Case presentation:**

The case of a 68-year-old male with carcinoid tumor arising in the urinary bladder is presented. Transurethral resection of a polypoid small tumor 0.4 cm in diameter was performed. Immunohistochemical study using neuroendocrine markers allowed a straightforward diagnosis of a low-grade neuroendocrine carcinoma (carcinoid tumor) of the urinary bladder. Immunohistochemistry demonstrated calcitonin immunoreactivity in the most of the tumor cells.

**Conclusion:**

This tumor shows specific clinical, macroscopical and histological features and must be considered in the differential diagnosis of bladder neoplasms.

## Background

The occurrence of primary carcinoid tumor of the urinary bladder is extremely rare. Two cases of these neoplasms have been reported recently by Martignoni and Eble [1]. According to a critical review of the literature performed by these authors, only four [2-5] of twelve previously reported cases showed convincing evidence od neuroendocrine differentiation and might therefore be regarded as pure carcinoid tumors of the urinary bladder. We report a case of a primary calcitonin-producing carcinoid tumor of the urinary bladder.

## Case presentation

A 68-year-old man was admitted in January 2004 for a macroscopic hematuria. The patient had no relevant past medical or family history, and laboratory data were all within normal limits. A cystoscopic examination was performed and a transurethral resection of a tiny, sessile polypoid lesion of the bladder neck was carried out. Clinical evaluation included ultrasonography and computerized tomography (CT) scan. The patients was seen at last follow-up 14 months after diagnosis and he was completely negative.

**Figure 1:**
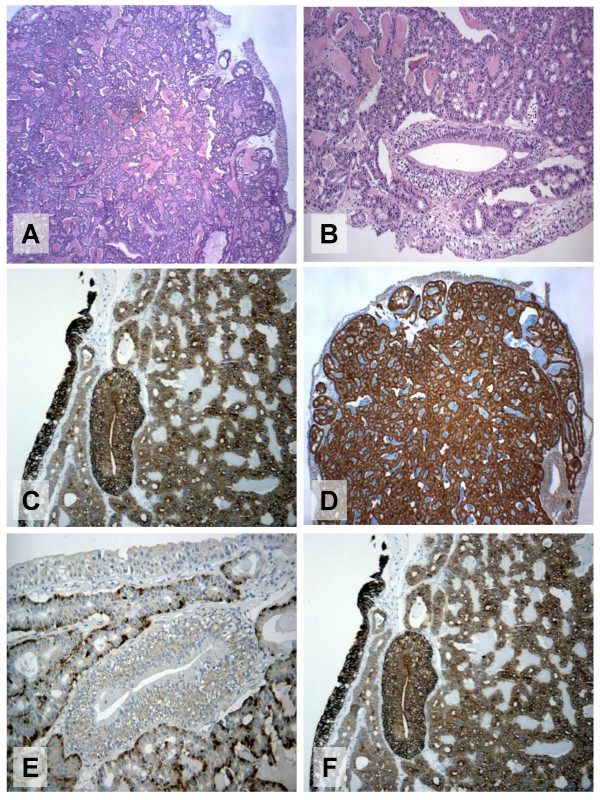
**Composite figure of carcinoid tumor of urinary bladder**. **A) **a glandular arrangement of tumor cells is evident. **B) **normal urothelium lined the tumor. A von Brunn nest entrapped by the tumor is seen. **C-D-E) **diffusely positive synaptophysin, chromogranin, and calcitonin immunostaining of tumor cells, respectively. **F) **urothelium and tumor cells strongly reacted with keratin 7.

Grossly, the tumor was a well-circumscribed, small ovoid nodule of 0.4 cm in diameter. Histologically a polypoid tumor appeared to be covered by slightly attenuated normal urothelium. The tumor filled-up the lamina propria showing a predominantly glandular arrangement (Fig 1A), with scanty anastomosing cords of cuboidal cells with fairly regular nuclei. Foci of von Brunn nests were seen at the periphery near the urothelium (Fig 1B). Mitoses and necrosis were not seen. A panel of immunostains, including antibodies against keratin 7, βHCG, chromogranin, synaptophysin, S-100 protein, NSE, serotonin, calcitonin, TTF-1, progesteron, and p53 protein was applied to representative sections of the tumor using the avidin-biotin complex technique (tab 1).

Immunohistochemically the tumor strongly reacted with synaptophysin (Fig 1C), chromogranin (Fig 1D), calcitonin (Fig 1E), keratin 7 (Fig 1F), and NSE. βHCG was positive in about 10% of the neoplastic cells. p53 protein, progesteron, S-100 protein, serotonin and TTF-1 were negative.

## Conclusion

The urinary bladder can be the site of tumors exhibiting various degrees of endocrine differentiation. It has become the most common site of extrapulmonary small cell undifferentiated carcinoma [6]. The spectrum of endocrine tumors of the urinary bladder has widened considerably in the past concerning both its morphological feature and its clinical behaviour. The better differentiated members of this group, carcinoid tumors, have an organoid arrangement, with trabecular and glandular patterns of growth, and its origin in the urinary bladder is extremely rare. To the best of our knowledge only 6 cases of primary carcinoid tumor of the bladder have been reported [1-5].

The present case shows clinically and microscopically the same features showed by two cases of carcinoid of urinary bladder reported recently by Martignoni and Eble [1]. Their review of the literature disclosed 4 previously reported pure carcinoid tumors of the urinary bladder. These 6 cases and the present case show a striking clinical and pathological overlapping, as shown in tab 2.

All 6 cases presented with hematuria, most of the patients aged of seven decade. All tumors were small, polypoid nodules at cystoscopy and most of them were localized in the neck of the bladder or trigone. All previously reported 6 tumors and the present case show a glandular architecture. Previous study, and our results have demonstrated the neuroendocrine differentiation. Strikingly the present case showed, immunohistochemically, a strongly reactivity with calcitonin. To the best of our knowledge this is the first case of a well-differentiated neuroendocrine carcinoma of the urinary bladder reported positive with this neuropeptide.

Some tumors could simulate carcinoid of the bladder, in particular the nested variant of transitional cell carcinoma; differences in the cytologic features and immunohistochemistry should establish the diagnosis. The distinction between carcinoid tumor and inverted papilloma with glandular differentiation can be very difficult, however strong staining for neuroendocrine markers support the diagnosis of carcinoid tumor. Like the classic carcinoid tumors of the appendix, small bowel, or lung, all of the carcinoid tumors of the bladder are histologically and clinically not aggressive.

In conclusion, we here add another case of carcinoid tumor of the urinary bladder to the existing literature, this tumor shows specific clinical, macroscopical and histological features and must be considered in the differential diagnosis of bladder neoplasms.

## Competing interests

The author(s) declare that they have no competing interests.

## Authors' contributions

MM participated to write the manuscript

VA operated the patient and participated to draft the manuscript

CM carried out imunohistochemical study

GN operated the patient and participated to draft the manuscript

GDR participated in the design of the study

LI participated in the design of the study

## Pre-publication history

The pre-publication history for this paper can be accessed here:



## Figures and Tables

**Table 1:** Primary antibodies used for immunophenotyping

Antibody	Manufacturer	Dilution	Method
Keratin 7	DAKO, Carpinteria, CA, USA	1:200	ABC
Chorionic Gonadotropin	DAKO, Carpinteria, CA, USA	1:200	ABC
Chromogranin A	DAKO, Carpinteria, CA, USA	1:50	ABC
Synaptophysin	DAKO, Carpinteria, CA, USA	1:20	ABC
S-100	DAKO, Carpinteria, CA, USA	1:200	ABC
NSE	DAKO, Carpinteria, CA, USA	1:8000	ABC
Serotonin	DAKO, Carpinteria, CA, USA	1:100	ABC
Calcitonin	Santa Cruz Biotechnology Inc, Santa Cruz, CA	1:100	ABC
Thyroid transcriptional factor	DAKO, Carpinteria, CA, USA	1:100	ABC
Progesteron	DAKO, Carpinteria, CA, USA	Ready to use	ABC
p-53 protein	DAKO, Carpinteria, CA, USA	1:50	ABC

**Table 2:** Clinicopathologic profiles of patients with carcinoid tumor of the urinary bladder.

**Author/Year/Reference**	**Age/Sex**	**Size (cm)**	**Symptoms**	**Cystoscopy**	**Location**	**Microscopy**
Colby 1980 (2)	30/M	0.3	Hematuria	Polypoid	Neck	Glandular
Walker et al 1992 (4)	62)F	1.2	Hematuria	Polypoid	Trigone	Glandular
Stanfield et al 1994 (5)	54/F	0.9	Hematuria	Polypoid	Neck	Glandular
Burgess et al 2000 (3)	61/F	0.3	Hematuria	Polypoid	Trigone	Glandular
Martignoni et al 2003 (1)	69/M	0.3	Hematuria	Polypoid	Neck	Glandular
Martignoni et al 2003 (1)	47/M	0.7	Hematuria	Polypoid	Neck	Glandular
Insabato et al (present study)	68/M	0.4	Hematuria	Polypoid	Neck	Glandular

## References

[B1] Martignoni G, Eble JN (2003). Carcinoid tumors of the urinary bladder. Immunohistochemical study of 2 cases and review of the literature. Arch Pathol Lab Med.

[B2] Colby TV (1980). Carcinoid tumor of the bladder. Arch Pathol Lab Med.

[B3] Burgess NA, Lewis DC, Matthews PN (1992). Primary carcinoid of the bladder. Br J Urol.

[B4] Walker BF, Someren A, Kennedy JC, Nicholas EM (1992). Primary carcinoid tumor of the urinary bladder. Arch Pathol Lab Med.

[B5] Stanfield BC, Grimes MM, Kay S (1994). Primary carcinoid of the bladder arising beneath an inverterd papilloma. Arch Pathol Lab Med.

[B6] Eble JN, Young RH (1997). Carcinoma of the urinary bladder: a review of its diverse morphology. Semin Diagn Pathol.

